# Synergistic protective effects of lycopene and N-acetylcysteine against cisplatin-induced hepatorenal toxicity in rats

**DOI:** 10.1038/s41598-021-93196-7

**Published:** 2021-07-07

**Authors:** Asmaa Elsayed, Ashraf Elkomy, Reda Elkammar, Gehan Youssef, Ehab Yahya Abdelhiee, Walied Abdo, Sabreen Ezzat Fadl, Ahmed Soliman, Mohamed Aboubakr

**Affiliations:** 1 Department of Pharmacology, Faculty of Veterinary Medicine, Benha University, 13736 Moshtohor, Toukh, Qaliobiya Egypt; 2 Department of Histology, Faculty of Veterinary Medicine, Benha University, 13736 Moshtohor, Toukh, Qaliobiya Egypt; 3 Department of Forensic Medicine and Toxicology, Faculty of Veterinary Medicine, Benha University, 13736 Moshtohor, Toukh, Qaliobiya Egypt; 4 Forensic Medicine and Toxicology Department, Faculty of Veterinary Medicine, Matrouh University, Matrouh, Egypt; 5 Pathology Department, Faculty of Veterinary Medicine, Kafrelsheikh University, Kafr Elsheikh, 33516 Egypt; 6 Biochemistry Department, Faculty of Veterinary Medicine, Matrouh University, Matrouh, Egypt; 7 Pharmacology Department, Faculty of Veterinary Medicine, Cairo University, Giza, 12211 Egypt

**Keywords:** Drug discovery, Nephrology

## Abstract

Cisplatin (CP) is one of the most frequently used chemotherapy agents. The objective of this design was to determine the ameliorative effect of lycopene (LP) and/or N-acetylcysteine (NAC) in rats with hepatic and renal toxicity induced by CP. Rats were divided randomly into 7 groups (7 rats/group): control vehicle group (saline only), the LP group (10 mg/kg, orally), the NAC group (150 mg/kg, orally), the CP group (7.5 mg/kg, IP on day 27), the LP-CP group, the NAC-CP group, and the LP-NAC-CP group. The activities of alanine aminotransferase (ALT), aspartate aminotransferase (AST), and alkaline phosphatase (APK), and levels of urea, creatinine, and lipids (cholesterol, triglycerides, and low-density lipoprotein-cholesterol) increased after CP injection in the serum. Moreover, CP decreased levels of protein, albumin, and HDL cholesterol. Meanwhile, malondialdehyde significantly increased with a decrease in reduced glutathione, superoxide dismutase, and catalase in the liver and kidney tissues. CP also induced some pathological lesions and increased the expression of caspase-3 in the liver and kidney tissues. Administration of LP and NAC alone or in combinations ameliorated hepatorenal toxicity and apoptosis induced by CP.

## Introduction

Cisplatin (CP) is one of the most frequently used chemotherapeutic drugs. Although cisplatin has well-known antineoplastic activity against multiple malignancies, adverse effects, mainly due to increased oxidative and cell apoptotic effects in different tissues, limit its administration^1,2^. CP is a highly active cytotoxic agent in cancer treatment^3^; however, nephrotoxicity limits its use^4^. CP administration leads to hepatic damage due to lipid peroxidation and oxidation leading to changes in liver biomarkers and antioxidant enzymes^5^. Many studies have reported CP-induced hepatotoxicity^6–9^ and nephrotoxicity^10–14^.

Lycopene (LP) is an acyclic carotenoid (a vitamin A derivative) with powerful and effective free-radical scavenging activity and anti-inflammatory, immunostimulant, antibiotic and anti-mutagenic effects^15,16^. LP is a red pigment that presents in high abundance in tomatoes and other red fruits. The chemical structure of lycopene contains many double bonds that have a major role in the scavenging reactive oxygen species (ROS)^17^. Consumption of tomatoes or tomato products is often associated with increased circulating lycopene levels and decreased oxidative damage to lipids, proteins, and DNA^18^. LP, a natural antioxidant, has antioxidant activity against several oxidative stress-mediated tissue injuries^19^. In *vitro* lycopene, antioxidant efficacy is up to 100 times more potent than vitamin E. In addition, LP has a chemo-preventive activity against certain forms of cancers^20^. The protective activity of LP against chemotherapeutic-induced hepatorenal damage has attracted considerable research activity in recent years.

N-acetylcysteine (NAC) is a medicinal and dietary supplement commonly used as a mucolytic agent to treat paracetamol overdose^21^. NAC is a pro-drug of L-cysteine, a precursor to glutathione (GSH). The oxidant-antioxidant balance can be modulated by NAC regulation of GSH levels in cells, inhibiting lipid peroxidation, and scavenging ROS^22,23^. NAC is capable of restoring the pro-oxidant/antioxidant balance and has been commonly used as an efficient antioxidant against oxidative stress both in vivo and in vitro^24,25^. CP has been used clinically for many diseases as a heavy metal chelator to protect against oxidative stress and prevent cell injury^26^. NAC has beneficial medicinal properties, including inhibition of carcinogenesis, tumorigenesis, mutagenesis, and tumor growth and metastases^27^.

The target of this design was to determine the protective effects of LP and/or NAC against hepatic and renal toxicity induced by CP in rats by exploring the biochemical, oxidative stress markers, and expression of caspase-3.

## Materials and methods

### Chemicals

CP (CAS No: 15663–27-1), (50 mg/ml parenteral administration) was bought from EIMC Pharmaceuticals Company (Cairo, Egypt). LP (CAS No: 502–65-8) was bought from Sigma Aldrich Company (Saint Louis, MO, USA). NAC (CAS No: 616–91-1) was bought from the South Egypt Drug Industries Company (SEDICO) (6 October City, Egypt). The analytical kits were bought from Bio-diagnostics Company (Giza, Egypt).

### Experimental rats and design

Forty-nine Wister Albino male rats with a weight of 190 ± 10 g were derived from the Egyptian Organization for Biological Products and Vaccines. All animals were housed at 25 ± 2° C, exposed to a 12:12 h light / dark cycle and given free access to water and commercial pellet, and left for 7 days for acclimatization prior to experiment. The experimental rats complied with the guidelines for the care and use of laboratory animals that were ethically approved by the Research Ethical Committee of the Faculty of Veterinary Medicine, Benha University, Egypt (Approval No. BUFVTM 03–03-21). Rats were divided randomly into seven groups (7 rats/group) as follows: the vehicle control group was given saline only once daily; the LP group received 10 mg/kg LP orally once daily^28^; the NAC group received 150 mg/kg NAC orally once daily^29^; the CP toxic control group received saline orally once daily and a single 7.5 mg/kg IP dose of CP on the 27th day of the experiment^30^; the LP + CP, NAC + CP, and LP + NAC + CP groups received LP, NAC, and/or CP as described above. Saline, LP, and NAC were administered for 30 days at 10 AM.

### Sampling and processing

Rats were anesthetized by isoflurane 1 day after the last treatment. Blood samples were collected from the retro-orbital plexus for separation of the serum (centrifugation at 1200 g for 15 min). The separated samples were stored at − 20 °C for further biochemical analysis. The biochemical parameters were Alanine aminotransferase (ALT), aspartate aminotransferase (AST), alkaline phosphatase (ALP), albumin, total protein, urea, creatinine, total cholesterol, HDL-cholesterol, triglycerides, and low-density lipoprotein (LDL). The previous biochemical tests were measured according data protocol provided by using commercial kits obtained from Biodiagnostic Company, Giza, Egypt. Finally, all dead rats (49) and remnants of samples were buried in the strict hygienically controlled properly constructed burial pit.

The livers and kidneys were quickly excised, washed with saline (0.9% NaCl in distilled water), and perfused with ice-cold 50 mmol/L sodium phosphate-buffered saline (100 mmol/L Na_2_HPO_4_/NaH_2_PO_4_, pH 7.4) containing 0.1 mmol/L EDTA. The tissue samples were stored at -80 °C until used. The tissue samples were homogenized on ice using an electrical homogenizer where 1 g tissue was homogenized with 5 ml phosphate buffer pH 7.4. N-ethylmaleimide was added directly after homogenization to prevent oxidation of GSH. After homogenization, the homogenates have been centrifuged at 1200 × g for 20 min at 4 °C for separation of supernatants. These supernatants were used for the detection of biomarkers of oxidative stress. The measured oxidative stress biomarkers were malondialdehyde (MDA; Biodiagnostic, # MD 2529, Egypt), catalase (CAT, Biodiagnostic, # CA 2517, Egypt), superoxide dismutase (SOD; Biodiagnostic, # SD 2521, Egypt), and reduced glutathione (GSH; Biodiagnostic, # SD 2511, Egypt).

For histological and immunohistochemical analysis the remaining liver and kidney tissues were immediately preserved with ten percent neutral buffered formalin for 24 h. After that, the tap water was used for washing the samples then the samples immersion in ethyl alcohol serial dilutions. Then the specimens were embedded in paraffin and cut into Sects. (4 μm thickness). The cut sections were stained with hematoxylin and eosin for histopathological examination under a light microscope^31^. For immunostaining, sections of the liver and kidney tissues were deparaffinized and dehydrated sequentially in graded ethyl alcohol. Then, sections were autoclaved (121 °C for 5 min) in distilled water for achieving antigen retrieval. After that, the slides were immersed in 3% H_2_O_2_ for inactivation of the endogenous peroxidase. For reducing nonspecific reactions, the slides were blocked in 5% bovine serum albumin blocking reagent for 20 min after washing 3 times in PBS. The blocking slides were incubated with polyclonal anti-caspase 3 antibodies (Invitrogen, Cat# PA5-77,887, dilution 1/100) for overnight at 4 °C followed by incubation with avidin–biotin complex (ABC kit, Vector Laboratories) at 37 °C for 45 min. The reaction product was visualized by treatment with 3,3-diaminobenzidine tetrahydrochloride (DAB) and the slides were counterstained with Mayer’s hematoxylin.

### Statistical analysis

Data are represented as the mean ± SE. Data were analyzed by one-way ANOVA followed by Duncan’s post hoc test for multiple group comparisons using the statistical software package SPSS for Windows (Version 21.0; SPSS Inc., Chicago, IL, USA). Differences were considered statistically significant at P < 0.05.

### Ethical approval

The current study was approved by the Ethical Committee for the live animals sampling at the Faculty of Veterinary Medicine, Benha University, Egypt.

### Consent for publication

Not applicable.

## Results

Cisplatin made an induction for hepatoxicity and nephrotoxicity that indicated by the elevated serum levels of the liver and kidney biomarkers (Table 1). AST, ALT, and ALP activities and concentration of creatinine, urea, cholesterol, triglycerides, and LDL cholesterol were substantially increased as a result of CP treatment compared to those of the control rats. Also, CP reduced the serum concentrations of total protein, albumin, and HDL cholesterol. On the other side, the case is different where these parameters were significantly reduced in the CP treated rats with LP, NAC, or combination treatment (LP and NAC) compared to the CP group. Notably, these parameters were significantly decreased when CP-intoxicated rats were treated with both LP and NAC relative to treatment with LP or NAC alone. The values were significantly lower compared to the controls. Thus, a combination of LP and NAC indicated better protection from hepatorenal damage caused by CP than either alone.

**Table 1:** Effects of LP, NAC, and/or CP on serum biochemical parameters (n = 7).

Parameters	Control	LP	NAC	CP	LYP + CP	NAC + CP	LP + NAC + CP
AST (U/L)	73.48 ± 0.79^C^	72.04 ± 0.73^c^	74.44 ± 1.16^c^	149.56 ± 4.29^a^	88.90 ± 1.53^b^	85.25 ± 2.60^b^	71.76 ± 1.96^c^
ALT (U/L)	44.07 ± 1.32^d^	42.31 ± 0.62^d^	45.88 ± 2.12^d^	113.59 ± 2.95^a^	95.33 ± 1.05^b^	93.82 ± 1.08^b^	81.76 ± 1.34^c^
ALP (U/L)	122.23 ± 1.73^e^	121.52 ± 3.44^e^	117.31 ± 3.61^e^	347.65 ± 17.14^a^	288.90 ± 1.53^b^	252.40 ± 6.42^c^	171.76 ± 1.96^d^
T. Protein (g/dl)	8.54 ± 0.09^a^	8.27 ± 0.05^ab^	8.19 ± 0.04^ab^	6.22 ± 0.06^e^	7.12 ± 0.31^d^	7.54 ± 0.09^c^	8.09 ± 0.02^b^
Albumin (g/dl)	4.41 ± 0.09^ab^	4.37 ± 0.11^ab^	4.56 ± 0.08^a^	3.11 ± 0.02^d^	3.75 ± 0.06^c^	3.81 ± 0.05^c^	4.29 ± 0.03^b^
Creatinine (mg/dl)	0.71 ± 0.01^de^	0.67 ± 0.01^e^	0.69 ± 0.01^de^	1.58 ± 0.03^a^	0.98 ± 0.02^b^	0.87 ± 0.01^c^	0.76 ± 0.03^d^
Urea (mg/dl)	36.58 ± 0.68^e^	38.09 ± 0.91^e^	39.43 ± 0.73^e^	104.73 ± 1.89^a^	83.18 ± 2.05^b^	72.39 ± 1.66^c^	59.62 ± 1.46^d^
Cholesterol (mg/dl)	87.71 ± 1.01^d^	86.35 ± 1.08^d^	88.23 ± 1.27^d^	152.85 ± 1.73^a^	131.97 ± 1.64^b^	119.47 ± 1.95^c^	91.13 ± 1.97^d^
Triglycerides (mg/dl)	46.57 ± 0.87^e^	48.15 ± 1.10^e^	45.88 ± 0.85^e^	97.70 ± 0.98^a^	83.18 ± 2.01^b^	78.11 ± 1.84^c^	61.76 ± 1.48^d^
HDL-Cholesterol (mg/dl)	56.28 ± 1.03^a^	54.52 ± 1.45^a^	55.88 ± 0.85^a^	29.85 ± 0.87^d^	38.69 ± 2.06^c^	40.96 ± 2.19^c^	45.33 ± 1.25^b^
LDL- Cholesterol (mg/dl)	22.11 ± 1.39^d^	22.19 ± 2.03^d^	23.17 ± 1.78^d^	103.45 ± 2.94^a^	76.63 ± 3.22^b^	62.87 ± 3.09^c^	22.02 ± 1.36^d^

Data are expressed as the mean ± SE (n = 7). Different superscript letters in the same row indicate statistical significance at P ≤ 0.05.

Cisplatin (CP) at a single dose of 7.5 mg/kg (IP); lycopene (LP) at a dose of 10 mg/kg; N-acetylcysteine (NAC) at a dose of 150 mg/kg; aspartate aminotransferase (AST); alanine aminotransferase (ALT); alkaline phosphatase (ALP); high density lipoprotein (HDL); low density lipoprotein (LDL).

The effects of CP intoxication and treatment with LP, NAC, and their combination on MDA, reduced glutathione, and antioxidant enzymes in the liver and kidney tissues are shown in Tables 2 and 3, respectively. MDA levels increased significantly and CAT, SOD, and GSH levels in the liver decreased significantly in CP-intoxicated rats compared to control rats. LP and NAC treatments attenuated the effects of CP on MDA in the liver tissue, CAT, SOD, and GSH, but these values were still significantly different from control values. Combined LP and NAC treatment significantly improved oxidative damage caused by CP in hepatic and renal tissues compared to the LP or NAC treatments alone. These results were confirmed by the result of histopathology.

**Table 2:** Effects of LP, NAC, and/or CP on antioxidant parameters in hepatic tissues (n = 7).

Parameters	Control	LP	NAC	CP	LYP + CP	NAC + CP	LP + NAC + CP
MDA (nmol/g)	141.81 ± 1.36^e^	144.88 ± 2.43^e^	143.5 ± 2.08^e^	283.37 ± 3.92^a^	187.84 ± 3.03^c^	201.16 ± 3.04^b^	172.87 ± 5.18^d^
CAT (U/g)	3.22 ± 0.04^a^	3.28 ± 0.04^a^	3.19 ± 0.04^a^	2.31 ± 0.06^e^	2.69 ± 0.03^d^	2.88 ± 0.06^c^	3.03 ± 0.04^b^
SOD (U/g)	33.32 ± 0.72^a^	32.86 ± 0.70^a^	34.03 ± 0.64^a^	21.01 ± 0.27^d^	24.64 ± 0.85^c^	25.02 ± 0.52^c^	29.88 ± 0.70^b^
GSH (mg/g)	122.96 ± 3.16^a^	119.46 ± 3.03^a^	121.05 ± 2.87^a^	81.48 ± 2.61^d^	92.31 ± 0.90^c^	93.05 ± 0.52^c^	106.65 ± 2.95^b^

Data are expressed as the mean ± SE (n = 7). Different superscript letters in the same row indicate statistical significance at P ≤ 0.05.

Cisplatin (CP) at a single dose of 7.5 mg/kg (IP); Lycopene (LP) at dose of 10 mg/kg; N-acetycysteine (NAC) at a dose of 150 mg/kg.

Malondialdehyde (MDA); catalase (CAT); superoxide dismutase (SOD); reduced glutathione (GSH).

**Table 3:** Effects of LP, NAC, and/or CP on antioxidant parameters in renal tissues.

Parameters	Control	LP	NAC	CP	LYP + CP	NAC + CP	LP + NAC + CP
MDA (nmol/g)	73.49 ± 0.77^d^	70.76 ± 0.80^d^	71.28 ± 1.07^d^	143.07 ± 4.85^a^	117.31 ± 2.11^b^	122.95 ± 5.16^b^	94.68 ± 1.56^c^
CAT (U/g)	2.20 ± 0.04^bc^	2.30 ± 0.04^ab^	2.32 ± 0.02^a^	1.43 ± 0.03^e^	1.82 ± 0.02^d^	1.78 ± 0.03^d^	2.09 ± 0.03 ^c^
SOD (U/g)	16.52 ± 0.19^a^	17.08 ± 0.43^a^	16.45 ± 0.26^a^	3.50 ± 0.15^e^	6.21 ± 0.06^c^	5.41 ± 0.11^d^	7.16 ± 0.23^b^
GSH (mg/g)	80.96 ± 1.76^a^	78.20 ± 0.45^ab^	81.12 ± 1.04^a^	45.58 ± 1.15^e^	53.62 ± 0.89^d^	64.15 ± 1.07^c^	75.65 ± 0.86^b^

Data are expressed as the mean ± SE (n = 7). Different superscript letters in the same row indicate statistical significance at P ≤ 0.05.

Cisplatin (CP) at a single dose of 7.5 mg/kg (IP);; Lycopene (LP) at dose of 10 mg/kg ;N-acetycysteine (NAC) at a dose of 150 mg/kg.

Malondialdehyde (MDA); catalase (CAT); superoxide dismutase (SOD); reduced glutathione (GSH).

**Figure 1:**
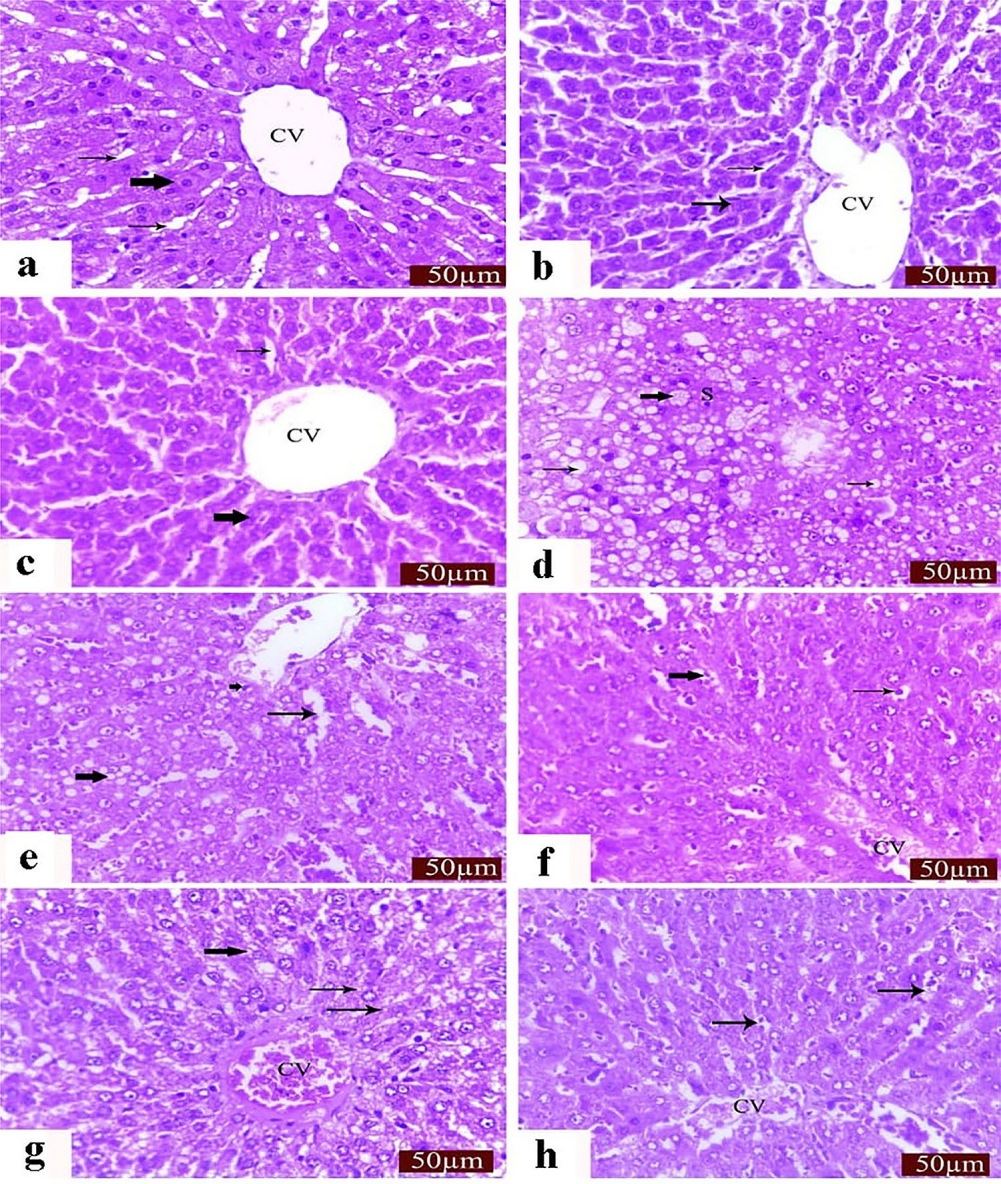
Histopathological changes in liver sections. (**a**)–(**c**) Normal organization of the hepatic cords (thick arrow), blood sinusoid (thin arrows), and central vein (CV) of the liver in the control (**a**), LP (**b**) and NAC (**c**) groups. CP-induced changes in the liver include severe degenerative changes, such as loss of normal hepatic cords arrangement, vacuolation (thin arrows), foamy appearance (thick arrow), and pycnotic nuclei of some hepatocytes (S). (**e**) CP-induced loss of normal cellular architecture of hepatic cords with severe hepatic vacuolation (thick arrow), dilated blood sinusoid (thin arrow), and pycnotic nuclei of hepatocytes (short arrow). (**f**) histological changes in the liver in the LP + CP group. Moderate effects in the liver include congested blood vessels (CV), dilated blood sinusoid (thick arrow), and condensation of nuclear chromatin of hepatocytes (thin arrow). (**g**) Histological changes in the liver in the NAC + CP group. Moderate effects in the liver include congested CV, hydropic degeneration (thin arrows), and degranulated cytoplasm of hepatocytes (thick arrow). (**h**) Histological changes in the liver in the LP + NAC + CP group. Mild effects in the liver include dilated blood vessels (CV) and blood sinusoid (arrows). Scale bar = 50 μm.

Histolopathological findings showed normal organized hepatocytes forming radiating hepatic cords around the central vein, with normal hepatic tissues including blood sinusoids and portal structures that observed in tissue sections from control saline, LP, and NAC-treated rats. In contrast, we observed severe alterative changes in the hepatic parenchyma, including loss of normal arrangement of hepatic cords, congested central vein and sinusoids, a foamy vacuolated cytoplasm and marked degenerative changes associated with severe nuclear pyknosis that noticed in sections of CP treated rats. Interestingly, LP, NAC, and their combination notably restored the usual hepatic architecture (Fig. 1).

**Figure 2:**
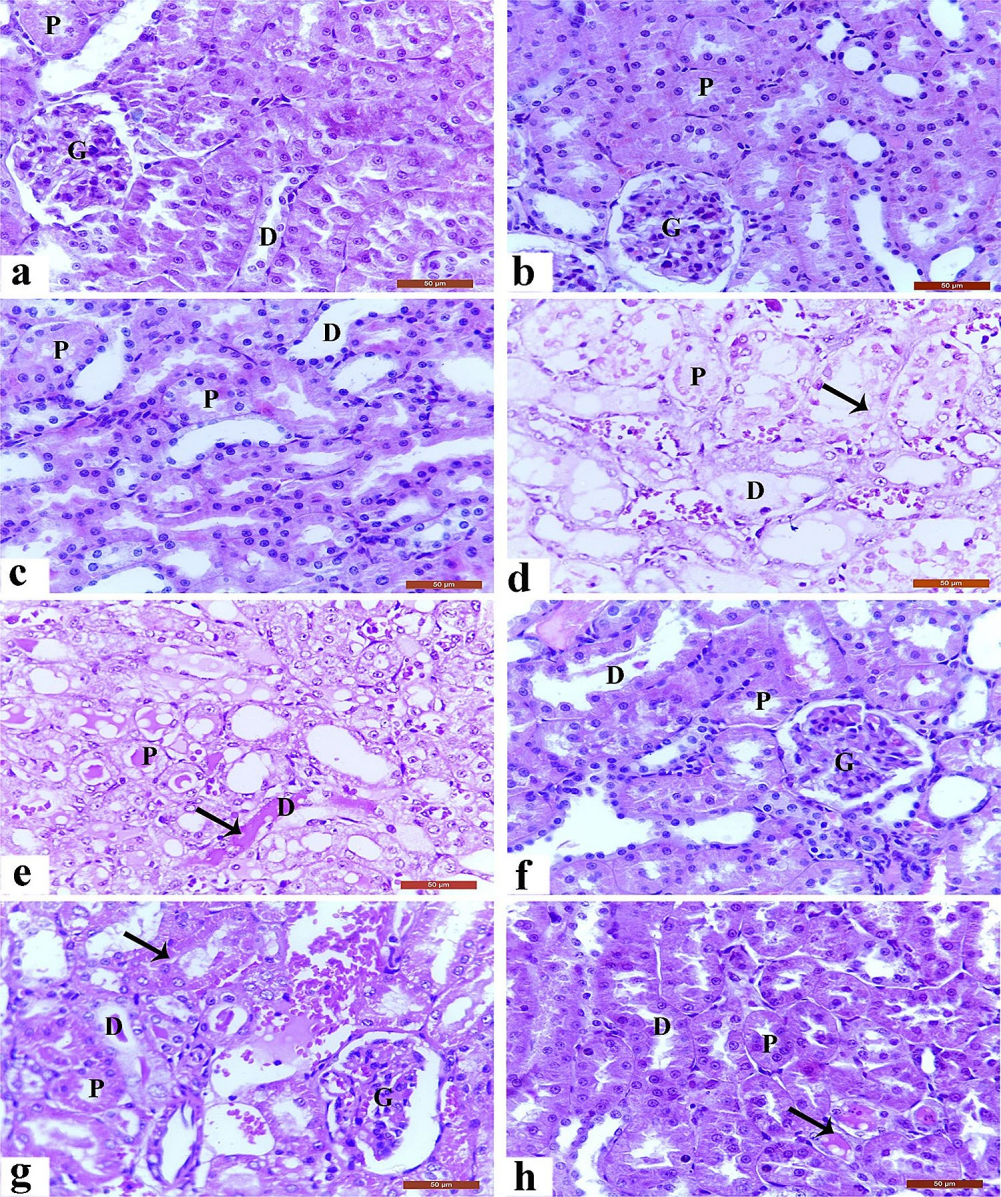
Histopathological changes in kidney sections. (**a**)–(**c**) Normal architecture of the renal cortex, renal corpuscle (arrow), glomerulus (G), proximal convoluted tubules (P), and distal convoluted tubules (D) in the control (**a**), LP (**1b**), and NAC (**d**) groups. (**d**) CP-induced severe degenerative changes 1in the renal tubule epithelial lining (**f**), congested inter-renal blood vessels (I), hydropic degeneration (thin arrow), and pycnotic nuclei of epithelial cells (thick arrow). (**e**) CP-induced loss of normal architecture in the renal tubules, with hyaline cast materials in the lumen of most tubules (thick arrows), degranulated cytoplasm in some epithelial cells (**d**), and desquamated cells (thin arrow). (**f**) Histological changes in the kidney in the LP + CP group. Moderate effects in the renal tubules and degenerated and sloughed epithelial cells (thick arrow). (**g**) Histological changes in the kidney in the NAC + CP group. Moderate effects include congested inter-renal blood vessels (C), hyaline cast materials in the lumen of some tubules (h), and necrosis in some epithelial cells (**e**). (**h**) Histological changes in the kidney in the LP + NAC + CP group. Mild effects include proteinaceous materials in the lumen of some tubules (thick arrows). Scale bar = 50 μm.

On the other side, Fig. 2 showed the normal architecture of the renal cortex, renal corpuscle, glomerulus, proximal convoluted tubules, and distal convoluted tubules of rats from the control, LP, and NAC groups. In contrast, CP-intoxicated rats exhibited severe nephrotic lesions associated with marked degenerative changes within the renal tubular epithelial lining, congested inter-renal blood vessels, hydropic degeneration, pyknotic nuclei in epithelial cells, and hyaline cast materials in the lumen of most tubules. Treatment with LP, NAC, or their combination prevented the histopathological kidney changes induced by CP.

**Figure 3:**
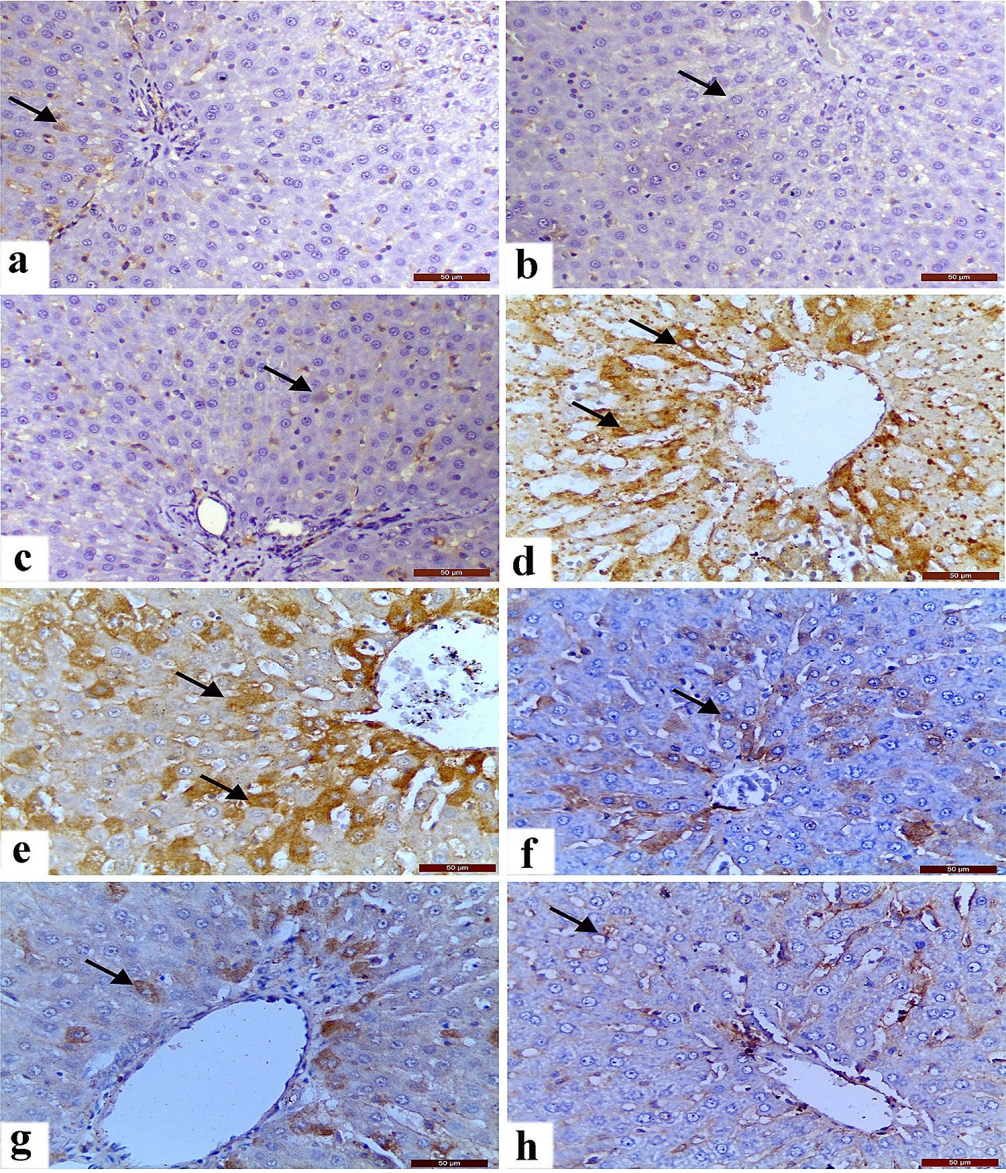
Changes in hepatic caspase-3 expression. (**a**)–(**c**) showed the negative immunostaining reactions in the Control (**a**), LP (**b**), and NAC (**c**) groups. (**d**) and (**e**) CP-induced changes showing severe immunostaining reaction. (**f**) Caspase staining in the LP + CP group showing moderate immunostaining. (**g**) Caspase staining in the NAC + CP group showing moderate immunostaining. (**h**) Caspase staining in the LP + NAC + CP group showing mild immunostaining. Scale bar = 50 μm.

**Figure 4:**
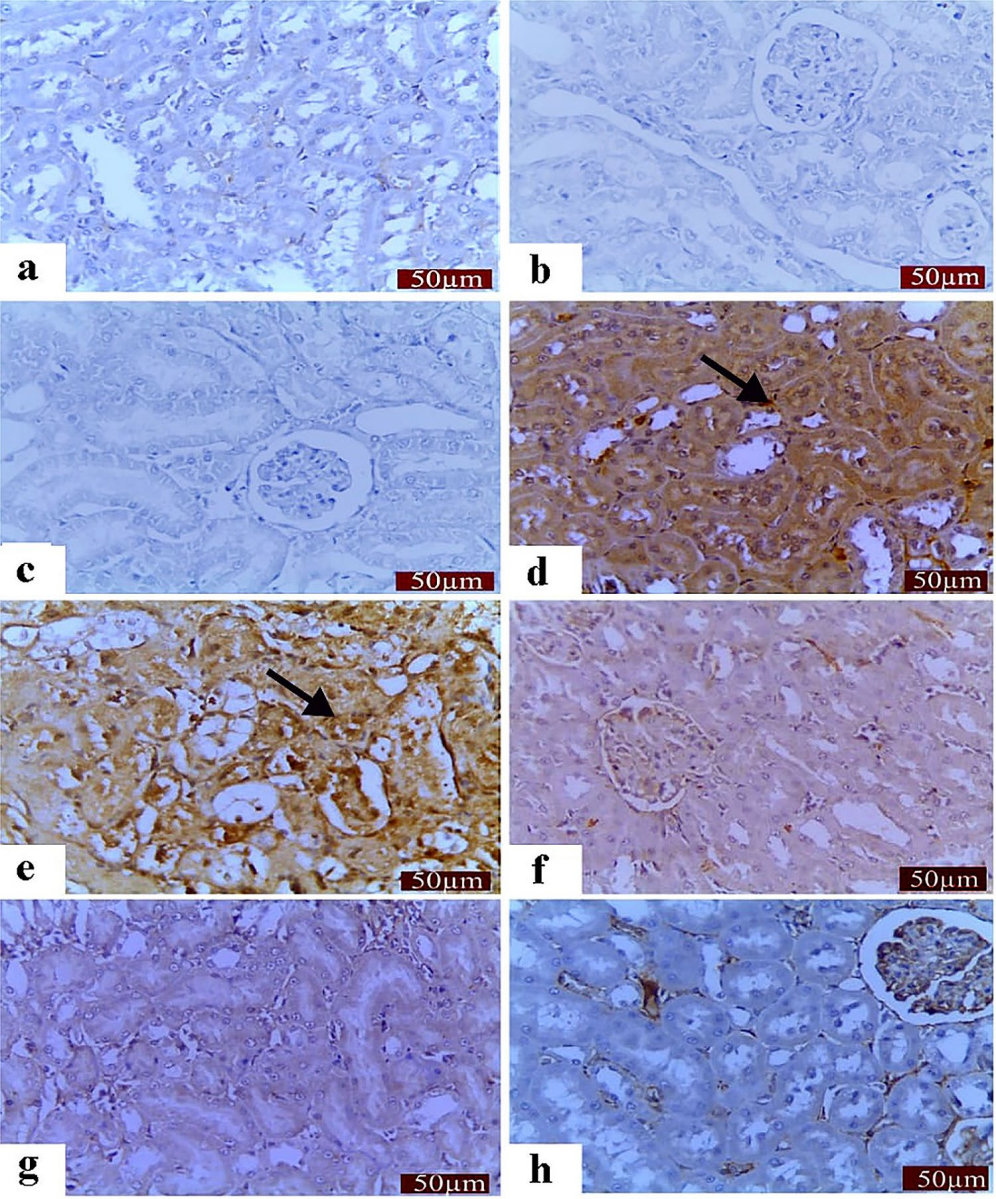
Changes in renal caspase-3 expression. (**a**)–(**c**) showed the negative immunostaining reactions. (**a**). Control group showing very mild caspase-3 immunostaining. Caspase-3 staining in the LP (**b**) and NAC (**C**) groups showing the negative immunostaining reaction. (**d**) and (**e**) treated group with CP showed severe immunostaining reaction. (**f**) Caspase staining in the LP + CP group showing moderate immunostaining. (**g**) Caspase staining in the NAC + CP group showing moderate immunostaining. (**h**) Caspase staining in the LP + NAC + CP group showing mild immunostaining. Scale bar = 50 μm.

Regarding results of the immunohistochemical, there was a dramatic up-regulation of caspase-3 either cytoplasmic or nuclear expression in the hepatic and renal tissues caused by CP (Fig. 3 and 4, respectively). On the other side, caspase-3 expressions slightly up-regulated in contrast to the control group were found in the LP + CP and NAC + CP groups. Moreover, the CP-mediated caspase-3 up-regulation reduced sharply by combined treatment of LP and NAC.

## Discussion

CP elicits anticancer effects by interacting with DNA and inducing programmed cell death. Multiple in vitro studies have demonstrated the cytotoxic effects of CP in different cell lines, but only a few in vivo studies have been performed^4,5,32–36^. Our findings are consistent with the in vivo results of other studies, including the involvement of oxidative stress and apoptotic mechanisms in CP-induced hepatorenal damage and the potential use of LP and NAC as protective agents against CP-induced injury.

Elevated activities of liver enzymes indicate cellular leakage and loss of functional hepatocyte integrity; the liver enzymes are released into the bloodstream when hepatocyte plasma membranes are impaired^1^. In this study, CP-induced hepatotoxicity was evidenced by significant alternations in serum liver enzymes (AST, ALT, and ALP). CP is taken up by the liver and accumulates in hepatocytes, causing cellular damage that eventually leads to increases circulating liver enzymes^8^. In addition, CP elevated creatinine and urea levels, in agreement with previous studies^1,17,37^. Elevated creatinine and urea levels are caused by reduced glomerular filtration rate. Moreover, Cayir et al.^38^ attributed the toxicity of the liver and kidney caused by CP to free radicals that generate in the cells of the liver and kidney, resulting in peroxidation of the lipid and consequently leads to oxidative stress that damage cells.

CP administration induced significant decreases in total circulating protein and albumin. This result was in agreement with Abuzinadah and Ahmad^39^. Following liver damage, CP intoxication reduces protein synthesis and alters the functional integrity of the kidney, leading to proteinuria and, eventually, to decreased circulating protein levels^40^.

Administration of CP resulted in significantly increased circulating cholesterol, triglycerides, and LDL-cholesterol, and decreased HDL-cholesterol. The liver plays a vital role in regulating plasma cholesterol levels. Thus, when hepatic dysfunction is induced by drug treatment, serum total cholesterol (TC) and LDL-cholesterol levels are increased^41^. The substantial rise in serum levels of TC, triglycerides (TG), and LDL-cholesterol following exposure of rats to cisplatin is likely due to the adverse effects of CP, leading to hepatocellular dysfunction and impaired lipid metabolism, in agreement with the findings of Akindele et al.^42^. The liver synthesizes TG and transforms TG into very-low-density lipoprotein (VLDL) cholesterol for transport to peripheral tissues and impairment of VLDL-C synthesis results in elevated TG levels^43^. A marked recovery from CP damage was observed in the LP and NAC-treated groups. In addition, NAC and LP have hypolipidemic effects^28,44^.

Concerning the oxidative stress/antioxidant parameters, MDA levels (increased lipid peroxidation) were significantly increased and antioxidants (CAT, SOD, and GSH) were significantly decreased in liver and kidney tissues after CP treatment. These results are in agreement with Abd El-Kader and Taha^11^, Abdel-Razek et al.^12^, and Elkomy et al.^1^. The ROS generation such as superoxide anions and hydroxyl radicals results in the mediation of oxidative stress and depletion of plasma antioxidants^39^.

LP inhibits lipid peroxidation by reacting directly with various ROS and by preventing mitochondrial damage induced by CP. Our results suggest that LP interferes with the oxidation of mitochondrial membrane lipids. LP inhibits lipid peroxidation as a chain breaker, free radical scavenger, and antioxidant modulator^45^.

The beneficial effects of the NAC are attributed to its role as a strong free radical scavenger. The free sulfhydryl group of NAC can react directly with electrophilic compounds, such as free radicals^46^. NAC also stimulates GSH synthesis and, thus, stimulates endogenous antioxidant activity^22^. The direct antioxidant activity and stimulation of endogenous antioxidant activity explain the ability of NAC to restore oxidative homeostasis in the liver and kidney in our study. The free radical scavenging of NAC can prevent disrupted renal blood flow after inferior vena cava occlusion^47^ and prevent the reduced renal vascular resistance caused by CP^48^. The effect of NAC on renal blood flow and vascular resistance in CP-intoxicated rats can explain the improvement in renal function observed in this study.

In addition to the ROS scavenging activity, LP and NAC restored the activities of SOD and CAT and the levels of GSH in CP-intoxicated rats. Therefore, we strongly suggest that LP and/or NAC-mediated improvements in the serum biochemical parameters tested in this study were mediated by ROS suppression and the up-regulation of antioxidant mechanisms against CP-induced oxidative injury.

LP is composed of carbon and hydrogen atoms (C_40_H_56_) with many double bonds that reduce the energy required for the delocalization of electrons providing a good source of hydrogen atom donation required to stabilize free radicals. Since LP is lipophilic, it is integrated with the lipid bilayer of the cell membrane allowing to abstract H atom from LP instead of unsaturated fatty acids halting CP-induced lipid peroxidation seen by a drastic reduction in MDA^49^.

NAC is a thiol donor with antioxidant properties. It is an excellent source of sulfhydryl groups and is converted in vivo into metabolites that stimulate glutathione (GSH) production, thereby maintaining intracellular GSH levels, enhancing detoxification, and acting directly as a free-radical scavenger^50^.

The histological and immunohistochemical observations of the current study were in harmony and confirmed the alterations of the biochemical and oxidant/antioxidant parameters among the experimental groups. CP treatment showed marked nephrotoxic effects, including cytoplasmic vacuolization in tubular epithelial cells and apoptosis as reported by Alhoshani et al.^4^. Severe degenerative changes in the renal tubules occurred after CP treatment, including hydropic degeneration, pycnotic nuclei, increased cytoplasmic vesicles, cytoplasmic vacuolization, necrosis, and apoptosis of tubular cells, and desquamation of necrotic epithelial cells, filling the tubular lumens and forming hyaline casts. These results are in agreement with the results of Perše and Večerić-Haler^51^. CP treatment induced expression of caspases-3, suggesting the occurrence of tubular epithelial cell apoptosis. These results confirm the results of Liu et al.^52^ and Miller et al.^53^, who demonstrated that CP was metabolically converted to a more potent toxin, which caused DNA injury and mitochondrial DNA and respiration damage. These toxin-induced changes lead to the activation of apoptotic pathways and the initiation of inflammatory responses. The CP-induced hepatotoxicity, indicated by sinusoidal dilation, congestion of blood vessels, and disorganized architecture of hepatic lobules, are consistent with the CP-induced effects reported by Elkomy et al.^1^.

NAC had no side effects in the liver or kidney, as manifested by the normal histology in these tissues. Our data confirmed that treatment with NAC had a protective effect against nephrotoxicity and hepatotoxicity, indicated by attenuation of the CP-induced degenerative changes in the liver and kidney. Treatment of rats with LP reduced the nephrotoxic and hepatotoxic effects of CP, as indicated by moderate histopathological findings in the liver and kidney. A combination of LP and NAC had a great prophylactic effect against CP-induced hepatotoxicity and nephrotoxicity. The rats treated with LP and NAC before CP treatment had only mild histopathological lesions of the liver and kidney and mild expression of caspase 3, indicating a lowered level of apoptosis. These results are consistent with the findings of Jiang et al.^54^, who considered LP an antioxidant drug that protects the liver and kidney from oxidative damage induced by CP. These results are also in agreement with Abdel-Wahab et al.^46^, who demonstrated that NAC attenuated CP-induced nephrotoxicity and restored proper kidney functioning, and with De Vries^55^, who indicated that NAC promoted liver detoxification. NAC may also reduce CP concentration in the kidney by increasing its renal excretion and/or preventing its accumulation in the renal tissue, as reported by Appenroth^56^. Zhao and Shichi^57^ demonstrated that the free sulfhydryl group can react directly with electrophilic compounds, such as free radicals, and Yalcin^22^ demonstrated that NAC can act as an antioxidant drug.

Pretreatment with LP and/or NAC protected against CP-induced hepatorenal toxicity, as shown by the improved biochemical parameters, oxidative stress markers, histopathology, and caspase-3 expression. A dietary combination of LP and NAC may enhance ROS scavenging capacity^17^. Our data revealed that co-administration of LP and NAC elicits a better protective effect against CP insult than their individual supplementations.

Also, other natural products protected against hepatorenal toxicity induced by CP as *Citrullus colocynthis*^30^, garlic oil^9^, thymoquinone^39^, and L-carnitine^1^.

## Conclusion

Overall, our data suggest that CP causes significant tissue damage in the liver and kidney due to oxidative stress and apoptotic mechanisms as evidenced by altered biochemical parameters and histopathological lesions. The combination of LP and NAC exhibits protective effects against CP-mediated damage in the liver and kidney. Moreover, LP and NAC, both individually or in combination, provided pronounced hepatorenal protection against CP-induced oxidative stress and apoptosis.

## Data Availability

The datasets used and/or analyzed during the current study are available from corresponding author on reasonable request.
